# Inulin Improves the Redox Response in Rats Fed a Diet Containing Recommended Copper Nanoparticle (CuNPs) Levels, While Pectin or Psyllium in Rats Receive Excessive CuNPs Levels in the Diet

**DOI:** 10.3390/antiox14060695

**Published:** 2025-06-08

**Authors:** Aleksandra Marzec, Ewelina Cholewińska, Bartosz Fotschki, Jerzy Juśkiewicz, Katarzyna Ognik

**Affiliations:** 1 Department of Biochemistry and Toxicology, Faculty of Animal Sciences and Bioeconomy, University of Life Sciences in Lublin, Akademicka 13, 20-950 Lublin, Poland; aleksandra.marzec@up.lublin.pl; 2 Division of Food Science, Institute of Animal Reproduction and Food Research, Polish Academy of Sciences, Trylińskiego 18, 10-683 Olsztyn, Poland; b.fotschki@pan.olsztyn.pl (B.F.); j.juskiewicz@pan.olsztyn.pl (J.J.)

**Keywords:** copper nanoparticles, inulin, pectin, psyllium, cellulose, redox status, blood, tissues

## Abstract

The study aimed to determine the effect of dietary inclusion of the recommended (6.5 mg Cu/kg diet) or double the recommended (13.0 mg Cu/kg diet) levels of copper nanoparticles (CuNPs) in combination with different types of dietary fibre on selected redox status indicators in the blood and tissues of male Wistar rats. Control groups were fed diets containing cellulose and a mineral mixture with standard or enhanced content of CuCO_3_. The experimental groups were fed a diet supplemented with CuNPs (6.5 or 13 mg/kg) and combined with various fibre types—cellulose, pectin, inulin, or psyllium. After the feeding period, rats’ organs were collected to assess selected indicators of redox status. The obtained results suggest that the addition of dietary fibre in the form of inulin may beneficially stimulate the response of the redox system in the conditions of CuNPs nutrition at the recommended dose, pectin, or psyllium in the case of an excessive supply of CuNPs in the diet. Thus, selecting the appropriate type of dietary fibre based on the CuNPs’ level in the diet may effectively protect the organism from the potentially harmful prooxidative effect of CuNPs, ultimately contributing to a favourable regulation of their metabolic impact in the body.

## 1. Introduction

Nanotechnology, a rapidly advancing scientific field, is finding increasingly broad applications across various sectors, including medicine, the food industry, electronics, and environmental protection [1]. Nanoparticles have also garnered growing interest among nutritionists due to their potential dietary use, which offers new avenues for enhancing health, primarily by improving nutrient bioavailability [2]. Copper (Cu) is an example of a micronutrient for which dietary supplementation is necessary to ensure the proper functioning of the body. However, the conventional, standardly used Cu inorganic forms have relatively poor absorption in the organism [3,4]. The existing animal studies indicate that incorporating copper nanoparticles (CuNPs) into the diet may significantly enhance the absorption and utilisation of copper within the body [3,4,5,6]. However, dietary inclusion of copper nanoparticles in animals may pose certain risks. The current literature suggests that CuNPs may exhibit some toxicity towards living organisms [5,7,8], which is likely attributable, to a significant extent, to their capacity for inducing oxidative stress [9]. It is hypothesised that in the gastrointestinal tract lumen, CuNPs may dissociate into ionic forms, which, by catalysing the Fenton reaction, lead to the generation of highly reactive oxygen species [10,11,12]. The resulting increasing oxidative stress may lead to the loss of cellular functionality through lipid peroxidation, metabolic enzyme dysfunction, damage to protein structures, and DNA mutations [13,14,15]. Our previous research confirmed that replacing conventional CuCO_3_ with CuNPs in rat diets can enhance lipid peroxidation processes and impair antioxidant defences in the lungs and kidneys [16]. Nonetheless, this replacement contributed to reduced lipid peroxidation in the brain and liver and decreased protein oxidation and nitration, as well as DNA oxidation and methylation in the blood of the studied animals [17]. Given that the inclusion of copper nanoparticles in animal diets can exert both positive and negative impacts on certain aspects of the organism’s redox status, it appears plausible that this effect could be additionally beneficially modulated through dietary fibre incorporation. This common dietary component has demonstrated antioxidant properties [18,19,20,21]. The available literature suggests that prebiotic fibres, such as inulin—and to a lesser extent, pectin and psyllium—promote the production of short-chain fatty acids (SCFAs) during fermentation in the colon, which may exert antioxidant effects [19,20]. Additionally, inulin supports the growth of beneficial gut bacteria that produce vitamins essential for properly functioning antioxidant enzymes [20]. Dietary fibre may also bind heavy metals and other ions, including Cu ions in the small intestine, which could positively regulate CuNPs’ bioavailability while reducing the free radicals generated within the organism [21,22,23,24]. The results of our previous studies align with these findings, demonstrating that dietary fibre supplementation strengthens the intestinal barrier in rats receiving CuNPs in their diet [25]. Furthermore, adding dietary fibres such as pectin, inulin, or psyllium to a diet containing CuNPs at concentrations twice the nutritional recommendation helps maintain copper homeostasis within the organism by significantly modifying the absorption and excretion of this trace element [6].

It was hypothesised that a dietary combination of CuNPs with various types of fibre—neutral cellulose (control), prebiotic inulin, viscous pectin, or swelling psyllium—would influence the redox response, thereby modulating the metabolic effects of CuNPs within the organism. The study aimed to test this hypothesis by evaluating the impact of dietary inclusion of the recommended (6.5 mg Cu/kg diet) or double the recommended (13.0 mg Cu/kg diet) levels of CuNPs in combination with different types of dietary fibre—cellulose, pectin, inulin, or psyllium—on selected redox status indicators in the blood and tissues of rats. In terms of animal models, there is a notable similarity between the internal organs of rodents and humans, particularly regarding redox status. While in vivo data on metal nanoparticles derived from rats may not be directly applicable to humans, it is important to emphasise that this new information enhances our understanding in the field.

## 2. Materials and Methods

The study presented in this paper is part of a large research project aimed at determining the effects of the dietary combination of CuNPs with various fibre types—cellulose, inulin, pectin, or psyllium on multiple aspects of the biological response in rats. Therefore, the experiment’s design and the procedures used have previously been published in scientific articles [6,26,27,28].

### 2.1. Materials’ Characterisation: Copper Nanoparticles and Fibre Types

Commercially available copper nanoparticles (CuNPs) supplied by SkySpring Nanomaterials, Inc. (Houston, TX, USA) were used in the study. These CuNPs were selected because of their well-documented stability and clearly defined physicochemical properties, including a purity of 99.9% trace metal basis, the appearance of addle brown nanopowder, an aerodynamic Particle Size (APS) of 40–60 nm, a specific surface area (SSA) of ~12 m^2^/g, a spherical morphology, bulk density of 0.19 g/cm^3^, and true density: 8.9 g/cm^3^, without the presence of additional coatings, surfactants, or surface modifiers. The experiment used the same CuNPs as those used in our previous studies [4,6,16,17,26,27,28]. CuCO_3_, serving as the control source of dietary copper, was obtained from Merck KGaA (Darmstadt, Germany). α-Cellulose (Sigma, Poznań, Poland) was used as the control dietary fibre. The experimental diets included the following fibre sources: pectin (PectinE 440(I), Brouwland, Beverlo, Belgium), inulin (Frutafit Tex, Sensus, ’s-Hertogenbosch, The Netherlands), and psyllium (Psyllium husk powder, NaturaleBio, Rome, Italy).

### 2.2. Animal Study Protocol and Diet Composition

All procedures involving animals were carried out following Polish regulations on animal experimentation and ethical standards, and were fully compliant with Directive 2010/63/EU of the European Convention for the Protection of Vertebrate Animals for Experimental and Other Scientific Purposes [29]. The Local Ethics Committee for Animal Experiments in Olsztyn approved the experimental protocol (Approval No. 19/2021; Olsztyn, Poland; Approval date: 17 March 2021). The protocol containing the research questions, experimental schema with the in vivo study design, and analysis plan was submitted to the reviewers of the National Science Center (Kraków, Poland) for evaluation, and was subsequently approved and funded.

The study involved one hundred healthy outbred male Wistar rats Cmdb:Wi, obtained from a certified breeding facility (breeder register 051, Institute of Animal Reproduction and Food Research PAS, Olsztyn, Poland). After a two-week acclimatisation period, they were randomly divided into ten groups, each consisting of ten rats. The environment included a 12 h light–dark cycle, a temperature maintained at 22 ± 1 °C, a relative humidity ranging from 45% to 65%, and 15 air changes per hour. Random numbers were created using Microsoft Excel’s standard = RAND() function. In the room with the animals, the cages were placed so that the same number of rats from a given group was placed in respective places on the cage rack (top, bottom, left side, right side). The rats were fed for 6 weeks on a standard semi-purified rat diet with two levels of CuNPs (the recommended level and double that level, i.e., 6.5 and 13 mg/kg diet, respectively) in combination with different types of dietary fibre. All diets have been prepared in our laboratory using high-end ingredients, including casein as the main protein source, rapeseed oil as a fat source, and maize starch as the main energy source (see Table 1 for details). In the control diets, CuCO_3_ was incorporated into the mineral mix at a standard and high level (6.5 and 13 mg/kg diet). In CuNP-supplemented groups, CuCO_3_ was excluded from mineral mixtures, and CuNPs were added to diets as an emulsion in rapeseed oil to ensure safe handling. The control dietary fibre was α-cellulose at 8% of the diet. Experimental fibres—pectin (viscous), inulin (prebiotic), and psyllium (bulking)—were included at 6%, replacing a portion of the cellulose. The experimental protocol consisted of 10 groups, *n* = 10 per group. The sample size was determined based on our own previous research. A single animal was considered an experimental unit.

The study was conducted following the ARRIVE guidelines [30], and all possible measures were taken to minimise the suffering of the animals used in the experiment. During the period of experimental feeding, in the event of adverse effects related to humane endpoints, i.e., cessation of diet intake for more than 2 days, making specific sounds as a pain signal for more than 1 h, the appearance of neurological symptoms (e.g., ataxia, impairment in maintaining a favorable body position), and the presence of blood in the feces for more than 1 day, a veterinarian (employed for these purposes at the institute) may make a decision of humane euthanasia using the method of gradual filling of the chamber with the animal with carbon dioxide or the method of dislodging the cervical vertebrae of a previously sedated animal. To the best of our knowledge, based on the literature and our previous experiments, the above symptoms should not be related to the experimental factors (fiber, nanoparticles), and there is only a minimal risk of their occurrence.

After 6 weeks of dietary treatments, rats were fasted for 8 h and then anaesthetised *i.p.* with ketamine and xylazine (K, 100/kg BW; X, 10 mg/kg BW). None of the animals were excluded from the experiment. It was assumed that for each experimental group, the criterion for removing an animal from the experiment was humane endpoints. The project manager was the only person who was aware of the animal’s allocation to a particular study group. Not all of the analysis contractors were acquainted with the treatment animal allocation. Blood was drawn from the caudal vena cava into both EDTA and heparinised tubes. Animals were subsequently sacrificed by cervical dislocation, and major organs such as the heart, lungs, jejunum, liver, pancreas, kidneys, spleen, and testes were collected. Blood plasma was obtained by chilling and centrifugation at 350× *g* for 10 min at 4 °C. Tissue homogenates were prepared by homogenising 1 g of each organ sample in 9 mL of phosphate-buffered saline (PBS), followed by centrifugation (3000× *g*, 10 min, 4 °C). Supernatants obtained from centrifuged homogenates and plasma samples were stored at −80 °C until further analysis.

**Table 1:** The composition of experimental diets administered to rats for 6 weeks (this table was also published in [6,26,27,28].

	C	CH	CN	CNH	PN	PNH	JN	JNH	SN	SNH
Casein ^1^	14.8	14.8	14.8	14.8	14.8	14.8	14.8	14.8	14.8	14.8
DL-methionine	0.2	0.2	0.2	0.2	0.2	0.2	0.2	0.2	0.2	0.2
Cellulose ^2^	8.0	8.0	8.0	8.0	2.0	2.0	2.0	2.0	2.0	2.0
Pectin					6	6				
Inulin							6	6		
Psyllium									6	6
Choline chloride	0.2	0.2	0.2	0.2	0.2	0.2	0.2	0.2	0.2	0.2
Rapeseed oil	8.0	8.0	8.0	8.0	8.0	8.0	8.0	8.0	8.0	8.0
Cholesterol	0.3	0.3	0.3	0.3	0.3	0.3	0.3	0.3	0.3	0.3
Vitamin mix ^3^	1.0	1.0	1.0	1.0	1.0	1.0	1.0	1.0	1.0	1.0
Mineral mix ^4^	3.5	3.5	3.5	3.5	3.5	3.5	3.5	3.5	3.5	3.5
Maize starch ^5^	64.0	64.0	64.0	64.0	64.0	64.0	64.0	64.0	64.0	64.0
Calculation:										
Cu from, mg/kg										
CuCO_3_	6.5	13	0	0	0	0	0	0	0	0
CuNPs	0	0	6.5	13	6.5	13	6.5	13	6.5	13

^1^ Casein preparation: crude protein 89.7%, crude fat 0.3%, ash 2.0%, and water 8.0%. ^2^ α-Cellulose (SIGMA, Poznan, Poland), the main source of dietary fibre. ^3^ AIN-93G-VM [31], g/kg mix: 3.0 nicotinic acid, 1.6 Ca pantothenate, 0.7 pyridoxine-HCl, 0.6 thiamin-HCl, 0.6 riboflavin, 0.2 folic acid, 0.02 biotin, 2.5 vitamin B-12 (cyanocobalamin, 0.1% in mannitol), 15.0 vitamin E (all-rac-α-tocopheryl acetate, 500 IU g^−1^), 0.8 vitamin A (all-trans-retinyl palmitate, 500,000 IU/g), 0.25 vitamin D-3 (cholecalciferol, 400,000 IU g^−1^), 0.075 vitamin K-1 (phylloquinone), 974.655 powdered sucrose. ^4^ In the experimental treatments with CuNPs, the MX was deprived of CuCO_3_ to keep the operator safe while preparing the experimental diets, and the CuNP preparation was added as an emulsion along with dietary rapeseed oil. Such a procedure was successfully applied in the previous experiments. ^5^ Maize starch preparation: crude protein 0.6%, crude fat 0.9%, ash 0.2%, total dietary fibre 0%, and water 8.8%.

### 2.3. Blood and Tissue Analyses

In blood plasma, levels of selected redox status indicators including superoxide dismutase (SOD), catalase (CAT), ceruloplasmin (Cp), total antioxidant status (TAS), malondialdehyde (MDA), 3-nitrotyrosine (3-NT), protein carbonyl derivatives (PCs), 8-hydroxydeoxyguanosine (8-OHdG), DNA repair enzymes—Apurinic/Apyrimidinic Endonuclease 1 (APE-1) and 8-oxoguanine DNA glycosylase (OGG1)—and markers of apoptotic cell death, caspase 3 (Casp3) and caspase 8 (Casp8), were measured. The measurements were performed using a commercially available enzyme-linked immunosorbent assays (ELISA) kit, following the manufacturer’s instructions (Shanghai Qayee Biotechnology Co., Ltd., Shanghai, China). Absorbance readings were taken at 450 nm using an ELISA microplate reader (Sunrise™, Tecan Group Ltd., Männedorf, Switzerland). For global DNA methylation analysis, DNA was first isolated from blood using the Blood Mini Kit column-based extraction system (A&A Biotechnology, Gdańsk, Poland), according to the manufacturer’s protocol. Then, global DNA methylation levels were subsequently assessed in the isolated DNA using commercial diagnostic kits provided by Sigma-Aldrich (Taufkirchen, Germany). The activities of the antioxidant enzymes superoxide dismutase (SOD) and catalase (CAT), along with the concentration of malondialdehyde (MDA) as an indicator of lipid peroxidation, were assessed in homogenates of the heart, lungs, jejunum, liver, pancreas, kidneys, spleen, and testes, following the procedure outlined by Ognik and Wertelecki [32].

### 2.4. Data Analysis and Statistics

Statistical analyses were performed using STATISTICA software, version 12.0 (StatSoft Corp., Krakow, Poland). A two-way ANOVA was conducted to evaluate the effects of two main factors: the CuNPs dose (L: 6.5 mg/kg and H: 13 mg/kg) and the type of dietary fibre (cellulose, pectin, inulin, and psyllium), followed by Duncan’s multiple range post hoc test. In addition, *t*-tests were applied to compare each group receiving the lower CuNP dose (L) with the control group C (fed 6.5 mg/kg Cu from CuCO_3_ and cellulose as the fibre source), and to compare groups receiving the higher CuNPs dose (H) with the control group CH (fed 13 mg/kg Cu from CuCO_3_ with cellulose). Differences were considered statistically significant at *p* ≤ 0.05. SEM, the pooled standard error of the mean, was calculated as the standard deviation for all rats divided by the square root of rat number, *n* = 100.

## 3. Results

### 3.1. One-Way ANOVA

#### 3.1.1. C vs. CN, PN, JN i SN

**Figure 1:**
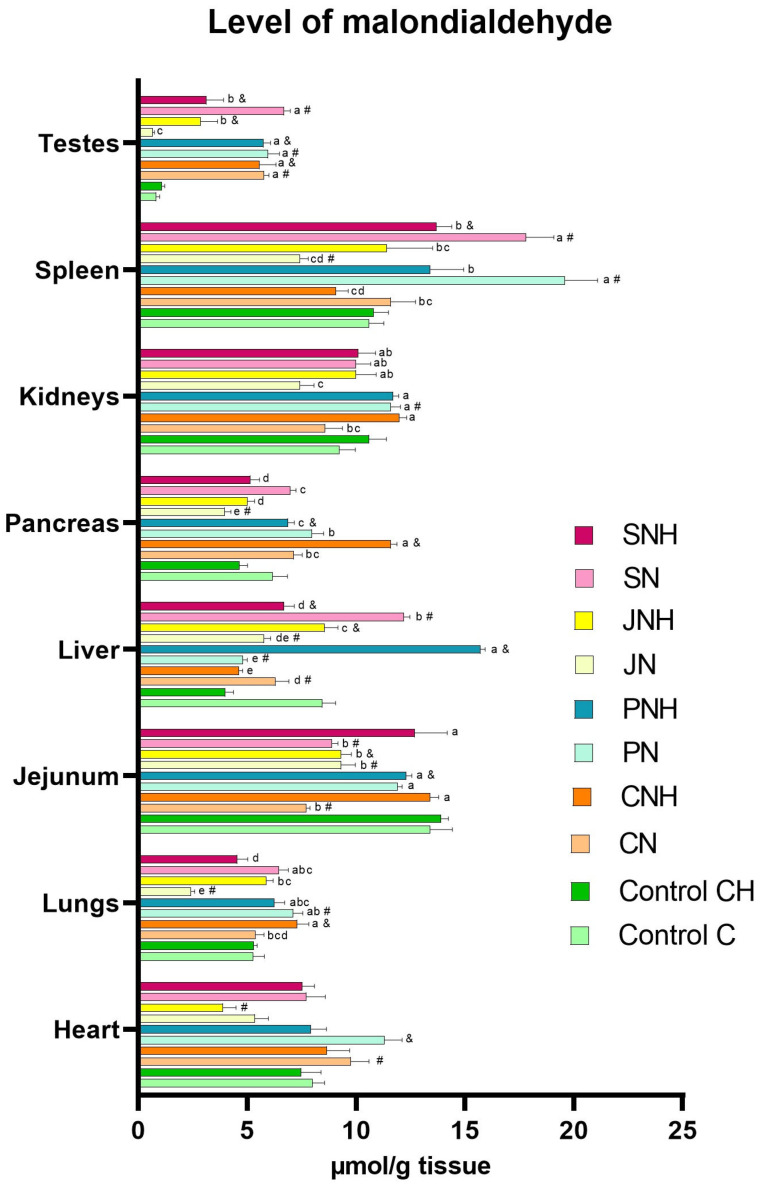
Level of malondialdehyde (MDA; µmol/g) in selected tissues in rats fed experimental diets (groups C and CH: control diets with standard (6.5 mg/kg) and enhanced (13 mg/kg) copper from CuCO_3_, with 8% cellulose as fibre; groups CN and CNH: diets supplemented with CuNPs (6.5 and 13 mg/kg) plus 8% cellulose; groups PN and PNH: diets supplemented with CuNPs (6.5 and 13 mg/kg), with fibre from 2% cellulose and 6% pectin; groups JN and JNH: diets supplemented with CuNPs (6.5 and 13 mg/kg), with fibre from 2% cellulose and 6% inulin; groups SN and SNH: diets supplemented with CuNPs (6.5 and 13 mg/kg), with fibre from 2% cellulose and 6% psyllium). ^a–e^ Mean values within a column with unlike superscript letters are shown to be significantly different (*p* < 0.05); differences among the groups (CN, CNH, PN, PNH, JN, JNH, SN, SNH) are indicated with superscripts only in the case of a statistically significant interaction D × F (*p* < 0.05). Additionally, each experimental group fed CuNPs 6.5 mg/kg (CN, PN, JN, SN) was compared with the control C one with the aid of *t*-test (^#^ indicates a significant difference versus the C group); similarly, each experimental group fed CuNPs 13 mg/kg (CNH, PNH, JNH, SNH) was compared with the control CH one with the aid of *t*-test (^&^ indicates a significant difference versus the CH group).

**Figure 2:**
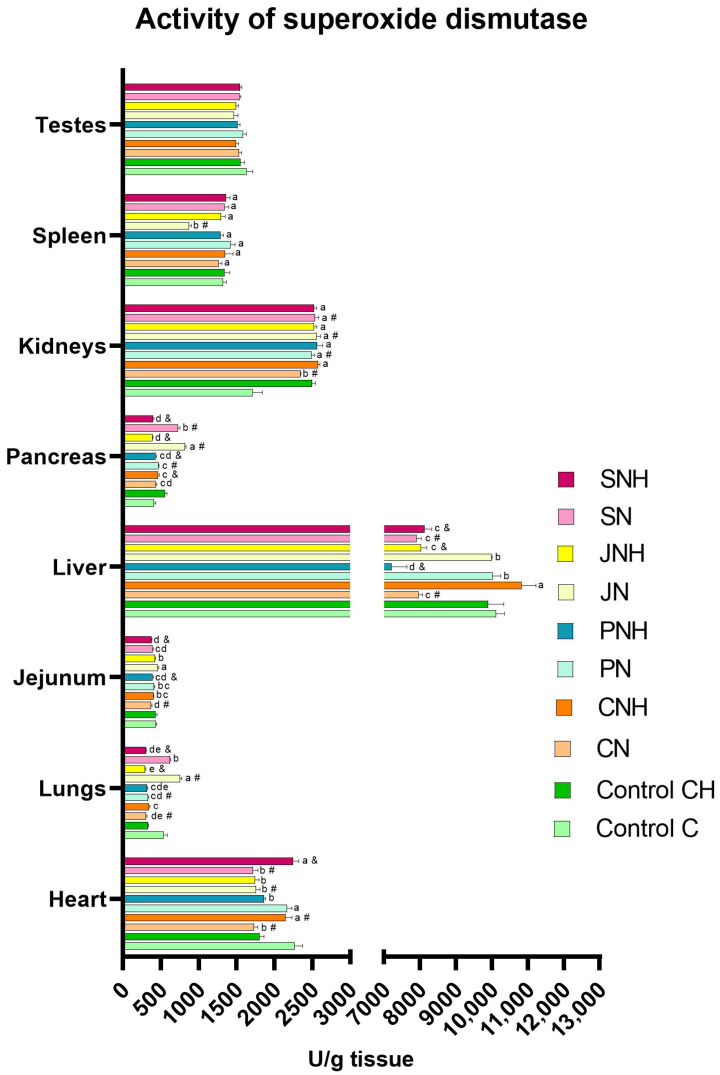
Activity of superoxide dismutase (SOD; U/g) in selected tissues in rats fed experimental diets (groups C and CH: control diets with standard (6.5 mg/kg) and enhanced (13 mg/kg) copper from CuCO_3_, with 8% cellulose as fibre; groups CN and CNH: diets supplemented with CuNPs (6.5 and 13 mg/kg) plus 8% cellulose; groups PN and PNH: diets supplemented with CuNPs (6.5 and 13 mg/kg), with fibre from 2% cellulose and 6% pectin; groups JN and JNH: diets supplemented with CuNPs (6.5 and 13 mg/kg), with fibre from 2% cellulose and 6% inulin; groups SN and SNH: diets supplemented with CuNPs (6.5 and 13 mg/kg), with fibre from 2% cellulose and 6% psyllium). ^a–e^ Mean values within a column with unlike superscript letters are shown to be significantly different (*p* < 0.05); differences among the groups (CN, CNH, PN, PNH, JN, JNH, SN, SNH) are indicated with superscripts only in the case of a statistically significant interaction D × F (*p* < 0.05). Additionally, each experimental group fed CuNPs 6.5 mg/kg (CN, PN, JN, SN) was compared with the control C one with the aid of *t*-test (^#^ indicates a significant difference versus the C group); similarly, each experimental group fed CuNPs 13 mg/kg (CNH, PNH, JNH, SNH) was compared with the control CH one with the aid of *t*-test (^&^ indicates a significant difference versus the CH group).

**Figure 3:**
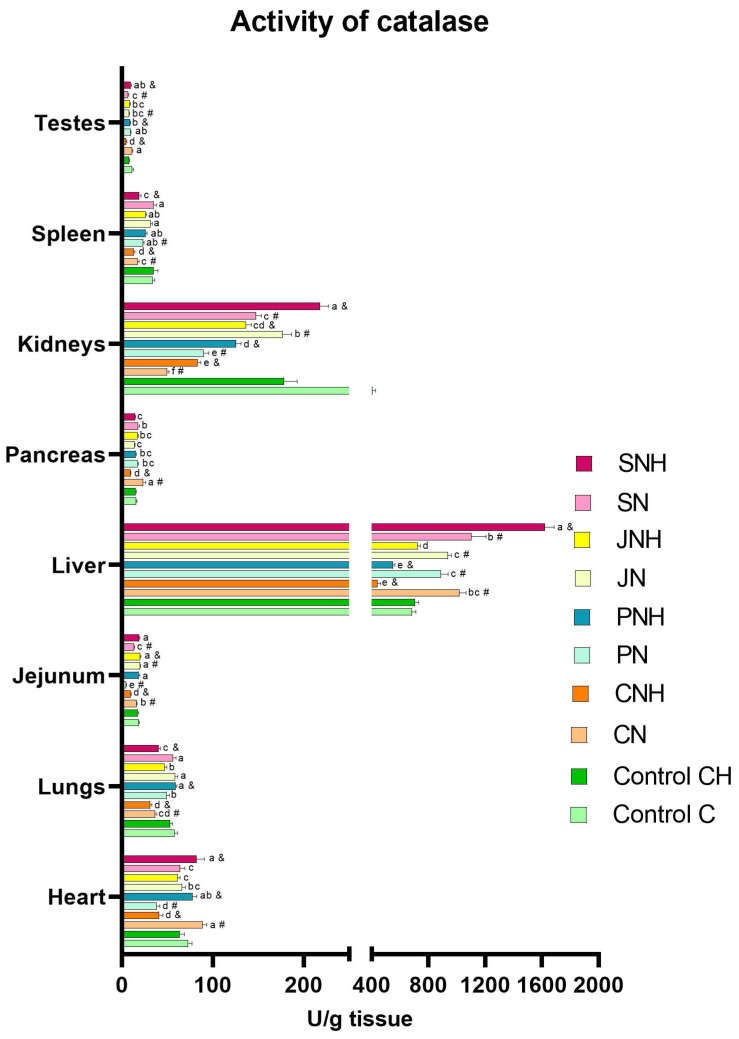
Activity of catalase (CAT; U/g) in selected tissues in rats fed experimental diets (groups C and CH: control diets with standard (6.5 mg/kg) and enhanced (13 mg/kg) copper from CuCO_3_, with 8% cellulose as fibre; groups CN and CNH: diets supplemented with CuNPs (6.5 and 13 mg/kg) plus 8% cellulose; groups PN and PNH: diets supplemented with CuNPs (6.5 and 13 mg/kg), with fibre from 2% cellulose and 6% pectin; groups JN and JNH: diets supplemented with CuNPs (6.5 and 13 mg/kg), with fibre from 2% cellulose and 6% inulin; groups SN and SNH: diets supplemented with CuNPs (6.5 and 13 mg/kg), with fibre from 2% cellulose and 6% psyllium). ^a–e^ Mean values within a column with unlike superscript letters are shown to be significantly different (*p* < 0.05); differences among the groups (CN, CNH, PN, PNH, JN, JNH, SN, SNH) are indicated with superscripts only in the case of a statistically significant interaction D × F (*p* < 0.05). Additionally, each experimental group fed CuNPs 6.5 mg/kg (CN, PN, JN, SN) was compared with the control C one with the aid of *t*-test (^#^ indicates a significant difference versus the C group); similarly, each experimental group fed CuNPs 13 mg/kg (CNH, PNH, JNH, SNH) was compared with the control CH one with the aid of *t*-test (^&^ indicates a significant difference versus the CH group).

The one-way analysis comparing the experimental groups CN, PN, JN, and SN to the control group C revealed a significant reduction in SOD levels in the blood plasma of all experimental groups (CN, PN, JN, and SN) compared to the control. Simultaneously, CAT levels were increased in the plasma of the CN, PN, and JN groups. In the plasma of rats in the PN and SN groups, a decrease in Cp levels alongside an increase in MDA levels compared to the control group C was noted. Additionally, in the blood plasma of rats from the PN, JN, and SN groups, higher levels of 8-OHdG were found than in the control group (C). Lower plasma PC levels were observed in the PN group compared to the C group. In turn, DNA methylation levels in the blood were decreased in the CN and SN groups vs. the C group. Lower levels of APE-1 and OGG-1 were observed in the blood plasma of rats from the SN group than in the control group C. A decreased level of APE-1 in the blood plasma was also observed in rats from the CN and JN groups. In the SN group, a decrease in the level of CASP3 and CASP8 in the blood was observed, whereas in the blood of rats from the JN group, only the level of CASP3 decreased compared to the C group (Table 2). In the CN group, an increase in MDA levels was recorded in the testes, while their levels in intestinal tissue and liver decreased compared to the control group (C). Relative to the control group (C), the MDA levels in the PN group increased in the heart, lungs, kidneys, spleen, and testes, but decreased in the liver. The JN group showed reduced MDA levels in the heart, lungs, intestinal tissue, liver, pancreas, and spleen vs. the C group. In contrast, the SN group exhibited increased MDA levels in the liver, spleen, and testes, with a concurrent decrease in the intestinal tissue compared to the control group (C) (Figure 1; see Supplementary Materials—Table S1 for details). In the CN group, an increase in SOD activity was noted in the kidneys; meanwhile, the decreased activity of this enzyme was detected in the heart, lungs, intestinal tissue, and liver compared to the C group. Relative to the control group (C), SOD activity in the PN group increased in the pancreas and kidneys but decreased in the lungs. The JN group showed increased SOD activity in the lungs, pancreas, and kidneys, alongside a decrease in this enzyme activity in the heart and spleen vs. the C group. In the SN group, increased SOD activity was observed in the pancreas and kidneys, while it decreased in the heart and liver compared to the control group (C) (Figure 2; see Supplementary Materials—Table S2 for details). In the CN group, CAT activity increased in the heart, intestinal tissue, liver, and pancreas but decreased in the lungs, kidneys, and spleen relative to the control group (C). Relative to control group C, CAT activity in the PN group increased in the liver but decreased in the heart, intestinal tissue, kidneys, and spleen. The JN group exhibited increased CAT activity in the intestinal tissue and liver, with lowered activity in the kidneys and testes vs. the C group. In the rats from the SN group, increased CAT activity was noted in the liver; meanwhile, its activity decreased in the intestinal tissue, kidneys, and testes compared to the control group (C) (Figure 3; see Supplementary Materials—Table S3 for details).

#### 3.1.2. CH vs. CNH, PNH, JNH i SNH

The one-way analysis comparing experimental groups CNH, PNH, JNH, and SNH to the control group CH indicated a significant reduction in SOD levels in the blood plasma of rats from the PNH, JNH, and SNH groups. Additionally, an increase in Cp levels in the blood plasma of the CNH and JNH groups was observed. Relative to the CH group, the levels of 3-NT and 8-OHdG in the blood plasma of rats from the PNH group were elevated. The MDA levels in the blood plasma increased in the JNH group but decreased in the SNH group compared to the control group (CH). Furthermore, blood samples collected from rats from the JNH and SNH groups exhibited higher PC levels than the CH group. There was also a reduction in the APE-1 level in the JNH group vs. the CH group, the OGG-1 level in the PNH and SNH groups vs. the CH group, and the CASP3 level in all experimental groups (CNH, PNH, JNH, and SNH) vs. the CH group (Table 2). MDA levels were higher in rats’ lungs, pancreas, and testes from the CNH group than in the rats from the control group (CH). Compared to the CH group, the MDA levels in the PNH group increased in the liver, pancreas, and testes but decreased in intestinal tissue. In the JNH group, rats exhibited increased MDA levels in the liver and testes while showing reduced levels in the heart and intestinal tissue relative to the control group (CH). In contrast, the SNH group demonstrated elevated MDA levels in the liver, spleen, and testes compared to the CH group (Figure 1; see Supplementary Materials—Table S1 for details). In the CNH group, increased SOD activity was noted in the heart; meanwhile, its activity was reduced in the pancreas compared to the control group (CH). Compared to the CH group, SOD activity in the PNH group decreased in the intestinal tissue, liver, and pancreas. Rats from the JNH group exhibited reduced SOD activity in the lungs, liver, and pancreas relative to the control group (CH). In turn, in the SNH group, SOD activity was increased in the heart but decreased in the lungs, intestinal tissue, liver, and pancreas (Figure 2; see Supplementary Materials—Table S2 for details). In the CNH group, CAT activity was reduced in the heart, lungs, intestinal tissue, liver, pancreas, kidneys, spleen, and testes relative to the control group (CH). Compared to the CH group, CAT activity in the PNH group increased in the heart, lungs, and testes, but its activity decreased in the liver and kidneys. In the JNH group, rats showed increased CAT activity in the intestinal tissue with reduced activity in the kidney compared to the CH group. The SNH group exhibited increased CAT activity in the heart, liver, and testes, while it decreased in the lungs, kidneys, and spleen compared to the CH group (Figure 3; see Supplementary Materials—Table S3 for details).

### 3.2. Two-Way ANOVA

The two-way ANOVA revealed the occurrence of significant interactions for MDA levels (*p* < 0.001), DNA methylation (*p* < 0.001), APE-1 (*p* = 0.037), and CASP3 (*p* = 0.019) in the blood (Table 2). Numerous interactions were also identified for MDA levels, as well as for SOD and CAT activities in various tissues (Figure 1, Figure 2 and Figure 3; see Supplementary Materials—Tables S1–S3 for details). The occurrence of interactions were noted for MDA levels in the lung (*p* < 0.001), intestinal tissue (*p* < 0.001), liver (*p* < 0.001), pancreas (*p* < 0.001), kidney (*p* = 0.020), spleen (*p* = 0.001), and testis (*p* < 0.001) (Figure 1; see Supplementary Materials—Table S1 for details), and for SOD and CAT activity in the heart (*p* < 0.001; both), lung (*p* < 0.001; both), intestinal tissue (*p* < 0.001; both), liver (*p* < 0.001; both), pancreas (*p* < 0.001; both), kidney (*p* < 0.001; both), and spleen (*p* < 0.001; both), and also CAT activity in the testis (*p* < 0.001) (Figure 2 and Figure 3; see Supplementary Materials—Tables S2 and S3 for details). The occurrence of the mentioned interactions indicates that the main effects had no significant influence on the parameters examined or were mutually cancelled out.

#### 3.2.1. Effect of CuNP Dose

The two-way ANOVA revealed that increasing the CuNPs level from 6.5 to 13 mg/kg diet did not significantly impact the redox parameters in blood and tissues (Table 2, Figure 1, Figure 2 and Figure 3; see Supplementary Materials—Tables S1–S3 for details).

#### 3.2.2. Effect of Fibre Type

Irrespective of the level of CuNPs, feeding rats a diet containing pectin, inulin, or psyllium resulted in a decrease in SOD levels in blood plasma (*p* < 0.001) compared to the control group receiving cellulose as a standard fibre source. Pectin or psyllium inclusion in the diet reduced Cp levels in blood plasma; meanwhile, inulin inclusion increased this indicator (*p* < 0.001) compared to rats receiving a diet containing only cellulose as a fibre source. Pectin or psyllium inclusion into the diet also elevated 3-NT levels (*p* = 0.033) in blood plasma compared to the control group. Furthermore, rats fed a diet supplemented with pectin had reduced PC levels (*p* < 0.001) in blood plasma, and psyllium supplementation to the diet resulted in elevated 8-OHdG levels (*p* = 0.028) in the blood plasma relative to the cellulose-only control group. Psyllium inclusion in the diet also significantly reduced CASP3 levels (*p* = 0.017) in the rats’ blood plasma compared to rats from the group that received a pectin-supplemented diet (Table 2). In the hearts of rats receiving an inulin-supplemented diet, a lower MDA level than in the control group was observed (Figure 1; see Supplementary Materials—Table S1 for details).

## 4. Discussion

The current experiment utilises Wistar rats (Cmdb:Wi) as they are a well-established model for nutritional and metabolic studies, particularly in examining the internal organs’ responses to nutritional interventions and the systemic metabolic changes associated with dietary supplementation. It is essential to acknowledge the limitations when extrapolating findings from animal studies to humans. These limitations arise not only from species differences but also from variations in dietary fibre and copper content, as well as the challenges of testing dietary nanoparticles in humans. Nevertheless, a substantial body of the literature supports the notion that the rat model offers significant advantages for investigating human health and disease in relation to dietary habits and environmental factors. The available literature indicates that the CuNPs introduced into the organism may induce oxidative stress [33,34,35]. The mechanism of the CuNPs’ prooxidant action seems to result primarily from the fact that, under the influence of the strong oxidants that occur in living cells, they can undergo oxidation processes to Cu^+^ and Cu^2+^ ions [3,36], which then enter the redox cycle [11,12]. In the presence of reducers such as ascorbic acid or glutathione, Cu^2+^ ions may reduce to Cu^+^ ions which, when reacting with hydrogen peroxide (H_2_O_2_) in the Fenton reaction, lead to the highly reactive hydroxyl radicals’ synthesis. Cu^+^ ions can also reduce molecular oxygen (O_2_), forming the superoxide anion radical (O_2_^•−^). Importantly, after reacting with H_2_O_2_ or O_2_, Cu^+^ ions re-oxidise to Cu^2+^, allowing them to re-enter the redox cycle, generating further reactive oxygen species (ROS) portions [11,12]. Due to their high reactivity and low specificity, these species can oxidise lipids, proteins, and nucleic acids, disrupting cellular function by compromising cell membrane integrity and protein functionality and causing DNA mutations that may contribute to cancer development [14,15].

Various antioxidant mechanisms have evolved in living organisms to mitigate the risk of such adverse oxidative changes induced by free radical activity. Among these, endogenous enzymatic antioxidant defence, including enzymes like superoxide dismutase (SOD) and catalase (CAT), plays a crucial role [37]. SOD catalyses the conversion of the aggressive superoxide radical (O_2_^•−^) to H_2_O_2_ and O_2_ [21], while CAT neutralises the resulting H_2_O_2_ to H_2_O and O_2_ [38]. The results of our study confirmed that replacing the standard Cu form (CuCO_3_) with CuNPs in rat diets influenced the enzymatic antioxidant defence, as evidenced by an increase in CAT levels and a simultaneous decrease in SOD levels in the blood. Potentially, this could indicate enhanced free radical synthesis due to the CuNPs’ action. The observed reduction in blood SOD levels may suggest the depletion of enzyme resources in response to increased superoxide anion (O_2_^•−^) generation in the presence of CuNPs. It is possible that the oxidative degradation of SOD could occur under experimental conditions, reducing its activity and stability, too. The increased CAT level may also indicate enhanced H_2_O_2_ synthesis due to the replacement of CuCO_3_ with CuNPs in the rats’ diet, which the body tries to combat by adjusting the enzyme activity to the current catalytic needs. However, it is worth noting that the observed changes in blood antioxidant enzyme levels, which suggest potential oxidative stress, were not accompanied by adverse changes in the total antioxidant status (TAS), biomarkers of protein oxidation (PC), lipid (MDA), and DNA (8-OHdG), or elevated markers of apoptotic cell death (CASP-3 and CASP-8). This suggests that the organism may adapt to the recommended level of CuNPs in the diet by modulating antioxidant enzyme activity to maintain proper redox status. Furthermore, the results of our study showed that replacing CuCO_3_ with CuNPs in the rat diet positively stimulated the antioxidant response in liver and small intestine cells, subsequently reducing lipid peroxidation, as indicated by decreased MDA levels. The small intestine is initially exposed to the highest CuNP concentrations due to direct contact with food content [27], while the liver serves as the main organ responsible for Cu metabolism and storage [39]. Both tissues are characterised by dense vascularisation and high metabolic activity [40,41], which favors exposure to potentially pro-oxidative CuNP action. Therefore, the obtained results seem to confirm that the dietary inclusion of CuNPs in the diet at the recommended dose does not generate excessive oxidative stress in the blood and tissues of rats.

However, it appears concerning that replacing CuCO_3_ with CuNPs in the rats’ diet led to reduced levels of APE-1 and DNA methylation in blood, potentially suggesting their negative impact on DNA stability and integrity. APE-1 (apurinic/apyrimidinic endonuclease 1) is a crucial enzyme in DNA repair through the base excision repair (BER) pathway. BER is initiated by DNA glycosylases, which identify and remove damaged bases, creating apurinic/apyrimidinic (AP) sites in the DNA chain. APE-1 then initiates the repair process by cutting the DNA backbone to remove AP sites and replacing them with correct nitrogenous bases, which helps maintain genome stability [42]. Therefore, the observed reduction in APE-1 levels in this study may suggest impairment of the DNA repair system or the increased degradation of this protein. Although this study did not demonstrate increased DNA oxidation as the increased production of 8-OHdG in the blood, it cannot be ruled out that extending the period of feeding rats with a diet containing CuNPs would lead to the complete inactivation of APE-1 and accumulation of oxidative DNA damage. Moreover, rats fed a diet containing CuNPs instead of CuCO_3_ showed a decrease in DNA methylation levels in their blood, which may lead to the deregulation of the expression of various genes, including those responsible for an oxidative stress response and DNA repair. The results of our study indicate that replacing the recommended level of CuCO_3_ in the rat diet with CuNPs does not negatively affect the oxidation of lipids and proteins in the blood and tissues. However, it may weaken DNA repair processes and reduce DNA methylation, significantly increasing the risk of mutations underlying numerous degenerative diseases.

The results of our study did not reveal an effect of doubling the level of CuNPs (13.0 mg Cu/kg diet) compared to nutritional recommendations (6.5 mg Cu/kg diet) in the rat diet on blood redox status indicators. However, it was found that the effect of CuNPs on blood redox status could be modulated by simultaneously introducing alternative dietary fibre sources. The results of the conducted studies showed a reduction in SOD levels in the blood of rats receiving a diet containing all tested dietary fibre forms—pectin, inulin, or psyllium—regardless of the CuNP level used. Meanwhile, none of these experimental treatments affected CAT activity in rat blood, which could indicate increased oxidative stress. It is hypothesised that the observed decrease in blood SOD levels could be due to a Cu deficiency in the body caused by the inclusion of different dietary fibre forms. Copper is an essential trace element for SOD1 and SOD3 synthesis because it is their cofactor with Zn. Cu’s presence in the active center of SOD is crucial for binding the super-oxide anion and its dismutation [43]. Consequently, a lack of Cu in the body may lead to limited SOD synthesis or impaired SOD antioxidant function, increasing the same the risk of oxidative stress and cellular damage. The available literature indicates that the presence of dietary fibre in the diet can decrease the absorption of trace elements, including Cu, from the gastrointestinal tract and impact their bioavailability and metabolism in the body [6,27,44,45,46]. The study by Krzysik [24] shows that adding pectin or cellulose as dietary fibre sources reduced the absorption of divalent ions from the diet, including Cu. The mechanism by which dietary fibre affects the absorption of Cu from the gastrointestinal tract seems to be closely dependent on the fibre’s type and physicochemical properties. The available literature indicates that pectin can chelate divalent ions via free hydroxyl groups, which may, in turn, reduce the amount of Cu available for absorption from the gastrointestinal tract [22,24,47]. Liu [33] and McRorie [48] also report that both pectin and psyllium have high viscosity and form a unique gel in the gastrointestinal tract, which the entrapment of Cu facilitates. This can physically hinder the dietary micronutrients’ contact with the absorptive surface of the intestines, thereby reducing Cu absorption [33,48]. Conversely, inulin seems to act oppositely to pectin and psyllium. It is a prebiotic that stimulates the growth of beneficial bacteria in the intestines, whose activity can affect mineral metabolism and absorption. Moreover, inulin fermentation produces short-chain fatty acids (SCFAs) such as butyrate, propionate, and acetate [49,50], which acidify the intestinal contents. Consequently, it may increase Cu solubility, thus enhancing its absorption from the gastrointestinal tract [50]. Nonetheless, irrespective of the type of dietary fibre used, this ingredient can accelerate intestinal transit, reducing the contact time of food contents with enterocytes of the small intestine, which may also limit the number of nutrients and minerals absorbed into the body [24,51]. The results of our previous studies have also confirmed that dietary fibre can limit the bioavailability of dietary Cu [6]. It has been shown that the addition of dietary fibre in the form of pectin, inulin, or psyllium to a diet with twice the CuNP content of dietary recommendations significantly increased Cu excretion and prevented its accumulation in the brain and muscles of rats [6]. In light of the above, it can be assumed that the observed decrease in blood SOD levels in our study may result from a Cu deficiency caused by its reduced absorption from the gastrointestinal tract due to supplementation with the tested alternative forms of dietary fibre. This hypothesis seems to be also supported by the decreased Cp levels in the blood of rats fed a diet with pectin or psyllium addition, regardless of the CuNP level. Ceruloplasmin is a glycoprotein that binds about 95% of Cu in blood plasma. Each molecule of ceruloplasmin can bind up to six atoms of this element. Thus, it is considered the most important blood Cu transport protein [52,53]. Furthermore, ceruloplasmin exhibits antioxidant properties, with effects similar to superoxide dismutase, although slightly weaker but more stable [54]. Ceruloplasmin is primarily synthesised in the liver, though small amounts can also be produced in the brain, placenta, kidneys, adipose tissue, and Sertoli cells [55]. This protein is sensitive to Cu deficiency, which quickly inhibits its synthesis [54]. Therefore, the reduced Cp levels in the blood of rats fed diets containing both CuNPs and pectin or psyllium may indicate a disturbance in Cu homeostasis due to its deficiency. The weakened antioxidant defence and increased oxidative stress of rats under a diet supplemented with pectin or psyllium seem to be confirmed by the increased level of 3-NT in the blood plasma, indicating the intensification of protein nitration processes. Additionally, the rats receiving psyllium in their diets, regardless of using CuNP levels, exhibited increased blood levels of 8-OHdG, suggesting enhanced nucleic acid oxidation. These observed adverse oxidative changes are likely due to a disturbance in Cu homeostasis, leading to a weakened antioxidant defence. Interestingly, however, inulin inclusions in the rats’ diet increased Cp levels in the blood, indicating its potentially beneficial effect on Cu metabolism in the body. This observed effect may be due to the antioxidant properties of inulin and improved Cu absorption in the gastrointestinal tract throughout the acidification of intestinal contents resulting from the fermentation of this type of fibre [54]. Moreover, rats who received a diet containing CuNPs, regardless of the dose, and inulin did not exhibit increased oxidative reactions of lipids, proteins, and nucleic acids in the blood. The conducted studies also showed that inulin inclusion in the diet, irrespective of the CuNPs’ supplementation level, reduced MDA levels in the heart. Therefore, it can be assumed that, unlike pectin and psyllium, the presence of inulin in the diet does not negatively affect Cu homeostasis and the blood antioxidant system, thereby enabling the effective protection of cells from oxidative processes.

The results of the redox status analyses of the internal organs conducted in this experiment did not show distinct effects of the main experimental factors, such as increasing the dietary CuNP level from the recommendation (6.5 mg Cu/kg) to twice the nutritional recommendation (13.0 mg Cu/kg) and replacing standard dietary fibre (cellulose) with alternative forms (pectin, inulin, or psyllium) in rat diets. Nevertheless, the obtained results allow us to notice certain relationships between the type of dietary fibre used and the level of CuNPs in the diet, in the context of the redox status of the tested organs. In rats fed a diet containing the recommended CuNP level and alternative dietary fibre, regardless of its form, the stimulation of the enzymatic antioxidant defence response was observed in the studied organs, with the effect being most beneficial in the case of inulin. Our previous research indicates that replacing the recommended level of CuCO_3_ with CuNPs in rats’ diets may intensify lipid peroxidation processes in the lungs and kidneys [17]. However, the current study showed that none of the analysed organs in rats supplemented with inulin and a standard dose of CuNPs in the diet exhibited increased lipid peroxidation. Interestingly, lipid peroxidation in the lungs, pancreas, and testes was even favorably inhibited, as evidenced by decreased MDA levels. In light of the above, it can be concluded that inulin effectively protects the body against the potentially toxic, pro-oxidant effects of CuNPs. In turn, pectin inclusion in the rats’ diets containing standard CuNP levels reduced lipid peroxidation in the liver but enhanced this process in the intestine and kidney. Psyllium supplementation in the rats’ diet containing a recommended level of CuNPs increased lipid peroxidation in the spleen and liver. Our previous studies indicate that replacing the recommended CuCO_3_ level with CuNPs in the rats’ diet attenuates lipid peroxidation processes in the liver. Therefore, it seems that the simultaneous inclusion of pectin or psyllium in the rat diet containing the recommended level of CuNPs is not sufficient to ensure the effective functioning of the antioxidant system, which may be probably related to the limitation of the amount of Cu absorbed from the gastrointestinal tract due to the strengthening of the intestinal barrier. Interestingly, the situation is markedly different with a twofold increase in dietary CuNPs. The most effective stimulation of antioxidant enzyme activity (SOD and CAT) in internal organs was observed in rats simultaneously receiving psyllium in their diets, which translated into reduced lipid peroxidation levels in the lungs, liver, pancreas, spleen, and testes. Nevertheless, rats from this treatment showed intensified lipid peroxidation in the small intestine, which may be related to the fact that this tissue was the first of all the examined tissues to be exposed to direct contact with the CuNPs introduced into the body with the diet and at the highest concentration. The elevated CuNP amount in the digestive contents could lead to the increased local concentration of reactive oxygen species, subsequently increasing lipid peroxidation in adjacent tissues. The results of our study indicate that, with excessive CuNPs in the diet, pectin addition could also favorably modulate the antioxidant system, protecting the pancreas and spleen from lipid oxidation. However, it was insufficient to shield the liver from CuNP-generated free radicals, as indicated by elevated MDA levels in this tissue. Among all the alternative fibres tested, inulin demonstrated the least protective effect against the prooxidative effects of excess CuNPs, as it did not reduce lipid peroxidation in any of the tissues examined and even exacerbated this process in the lungs, pancreas, kidneys, liver, and testes. It is highly likely that the improved redox status of the studied tissues through pectin or psyllium supplementation in the diet results from these fibres’ role in limiting the amount of CuNPs absorbed from the gastrointestinal tract, thereby reducing the generation of free radicals in the body. There is also a possibility that the antioxidant potential of the applied dietary fibers results from their direct beneficial effect on the gut microbiota composition and the increased production of short-chain fatty acids (SCFAs) during fiber fermentation. The literature data indicate that SCFAs exhibit strong antioxidant properties by enhancing the activity of antioxidant enzymes, inducing protective proteins, and activating the Nrf2–Keap1 signaling pathway [56]. Our previous research also confirmed that the inclusion of experimental types of dietary fiber in diets containing copper nanoparticles (CuNPs) significantly increased the activity of intestinal bacterial enzymes such as α-glucosidase, β-glucosidase, β-glucuronidase, and α-arabinofuranosidase, as well as the production of SCFAs in rats, with the most pronounced effect observed with pectin supplementation [57]. This suggests that functional fiber may mitigate the negative impact of CuNPs on the gut microbiota by supporting its metabolic activity, which in turn may translate into enhanced antioxidant protection of the organism. In turn, lipid oxidation in the tissues of rats receiving excess CuNPs in their diet, enhanced by inulin, is probably because this additive, due to its physicochemical properties, may support the absorption of Cu, or at least to a lesser extent than pectin and psyllium, inhibit its absorption. Consequently, this could increase Cu bioavailability in the body, raising the risk of enhanced reactive oxygen species generation.

## 5. Conclusions

In conclusion, replacing CuCO_3_ with CuNPs in the rat diet did not affect protein and lipid oxidation processes, but it deteriorated DNA stability and integrity in the blood by weakening the DNA repair system and reducing DNA methylation levels. However, this treatment favorably limited lipid peroxidation in the liver and small intestine. Regardless of the CuNPs level in the rats’ diet, inulin did not negatively impact redox status; meanwhile, pectin intensified protein nitration processes, and psyllium induced both protein nitration and DNA oxidation in the blood. Furthermore, adding pectin or psyllium to rats’ diets with standard CuNP content weakened the antioxidant defence of internal organs and intensified oxidative changes in tissues, likely due to the Cu deficiency resulting from increased Cu binding in the gastrointestinal tract. However, in the presence of excessive amounts of CuNPs in the diet, pectin or psyllium inclusion reduced the release of free radicals by CuNPs, thereby beneficially affecting the redox status of tissues. Meanwhile, inulin inclusion improved the redox status of tissues in rats fed the recommended CuNP level in the diet but worsened it when the dietary CuNP level was excessive, likely due to increased Cu absorption from the gastrointestinal tract in the presence of inulin. The obtained results suggest that the addition of dietary fibre in the form of inulin may beneficially stimulate the response of the redox system in the conditions of CuNP nutrition at the recommended dose, pectin, or psyllium in the case of an excessive supply of CuNPs in the diet. Thus, selecting the appropriate type of dietary fibre based on the CuNP level in the diet may effectively protect the organism from the potentially harmful prooxidative effect of CuNPs, ultimately contributing to the favourable regulation of their metabolic impact in the body.

## Figures and Tables

**Table 2:** Blood parameters in rats fed experimental diets (*n* = 10 per group) *.

	SOD	CAT	Cp	TAS	MDA	3-NT	PC	8-OHdG	DNA Methylation	APE-1	OGG1	Casp3	Casp8
	ng/mL	ng/mL	U/L	mmol/L	nmol/mL	ng/mL	nmol/mg Protein	ng/mL	%	ng/mL	pg/mL	ng/mL	ng/mL
Control C	36.6	27.1	164	0.902	1.49	14.8	4.75	6.93	1.46	180	493	434	102
Control CH	17.2	29.7	125	0.973	1.5 6	14.7	3.36	7.80	1.16	171	460	441	100
2-way ANOVA:													
CN	17.3 ^#^	31.7 ^#^	141	0.932	1.41 ^d^	14.6	4.82	7.93	0.853 ^b#^	167 ^a#^	454	402 ^a^	99.3
CNH	18.6	31.4	148 ^&^	0.974	1.50 ^cd^	14.6	4.20	7.76	1.52 ^a^	167 ^a^	460	387 ^ab&^	97.7
PN	8.31 ^#^	33.5 ^#^	131 ^#^	1.08	1.83 ^bc#^	15.8	3.35 ^#^	8.07 ^#^	1.28 ^a^	169 ^a^	471	411 ^a^	123
PNH	10.7 ^&^	32.2	125	0.943	1.64 ^cd^	18.4 ^&^	3.03	8.93 ^&^	0.915 ^b^	155 ^ab^	386 ^&^	335 ^c&^	101
JN	5.04 ^#^	31.9 ^#^	162	0.956	1.63 ^cd^	14.8	4.35	7.90 ^#^	1.59 ^a^	153 ^ab#^	428	347 ^bc#^	105
JNH	8.18 ^&^	29.9	158 ^&^	0.968	2.87 ^a&^	15.2	4.75 ^&^	8.45	0.909 ^b^	152 ^ab &^	408	350 ^bc&^	93.3
SN	6.99 ^#^	31.1	138 ^#^	0.954	2.07 ^b#^	16.8	5.29	9.05 ^#^	0.966 ^b#^	143 ^b#^	398 ^#^	330 ^c#^	77.9 ^#^
SNH	6.56 ^&^	31.0	129	1.09	1.36 ^d&^	17.2	5.01 ^&^	8.62	0.845 ^b^	162 ^a^	399 ^&^	350 ^bc&^	83.7
SEM	0.986	0.389	2.083	0.019	0.054	0.323	0.139	0.119	0.044	1.934	7.941	6.108	2.934
CuNPs dose (D)													
L (6.5 mg/kg)	9.42	32.1	143	0.981	1.74	15.5	4.45	8.24	1.17	158	438	373	101
H (13 mg/kg)	11.0	31.1	140	0.995	1.84	16.4	4.25	8.44	1.05	159	413	356	93.8
*p* value	0.065	0.242	0.239	0.724	0.197	0.268	0.480	0.406	0.110	0.737	0.137	0.132	0.309
Fibre type (F)													
C (cellulose)	18.0 ^a^	31.6	144 ^b^	0.953	1.45	14.6 ^b^	4.52 ^a^	7.84 ^b^	1.19	167	457	394	97.3 ^ab^
P (pectin)	9.53 ^b^	32.9	128 ^c^	1.01	1.74	17.1 ^a^	3.19 ^b^	8.50 ^ab^	1.10	162	428	373	112 ^a^
J (inulin)	6.61 ^c^	30.9	160 ^a^	0.962	2.25	15.0 ^ab^	4.55 ^a^	8.17 ^ab^	1.25	153	418	349	98.9 ^ab^
S (psyllium)	6.77 ^c^	31.0	133 ^c^	1.02	1.71	17.0 ^a^	5.15 ^a^	8.84 ^a^	0.906	153	399	340	80.8 ^b^
*p* value	<0.001	0.309	<0.001	0.513	<0.001	0.033	<0.001	0.028	0.015	0.024	0.090	0.004	0.017
Interaction D × F													
*p* value	0.488	0.837	0.196	0.117	<0.001	0.607	0.649	0.192	<0.001	0.037	0.192	0.019	0.445

* The dietary treatments used in the experimental feeding period: groups C and CH, fed a control diet with standard and enhanced Cu content in the mineral mixture (6.5 and 13 mg/kg from CuCO_3_, respectively) with 8% of cellulose as dietary fibre source; groups CN and CNH, fed diets with supplementation of CuNPs (6.5 and 13 mg/kg from Cu-nanoparticles, respectively), with 8% of cellulose dietary fibre source; groups PN and PNH, fed diets with supplementation of CuNPs (6.5 and 13 mg/kg from Cu-nanoparticles, respectively), with 2% of cellulose and 6% of pectin dietary fibre source; groups JN and JNH, fed diets with supplementation of CuNPs (6.5 and 13 mg/kg from Cu-nanoparticles, respectively), with 2% of cellulose and 6% of inulin dietary fibre source; groups SN and SNH, fed diets with supplementation of CuNPs (6.5 and 13 mg/kg from Cu-nanoparticles, respectively), with 2% of cellulose and 6% of psyllium dietary fibre source; L, treatment (*n* = 40) with dietary CuNPs (6.5 mg/kg dose); H, treatment (*n* = 40) with dietary CuNPs (13 mg/kg dose); C, treatment (*n* = 20) with cellulose as dietary fibre; P, treatment (*n* = 20) with pectin as dietary fibre; J, treatment (*n* = 20) with inulin as dietary fibre; S, treatment (*n* = 20) with psyllium as dietary fibre; ^a–d^ Mean values within a column with unlike superscript letters are shown to be significantly different (*p* < 0.05); differences among the groups (CN, CNH, PN, PNH, JN, JNH, SN, SNH) are indicated with superscripts only in the case of a statistically significant interaction D × F (*p* < 0.05). Additionally, each experimental group fed CuNPs 6.5 mg/kg (CN, PN, JN, SN) was compared with the control C one with the aid of *t*-test (^#^ indicates a significant difference versus the C group); similarly, each experimental group fed CuNPs 13 mg/kg (CNH, PNH, JNH, SNH) was compared with the control CH one with the aid of *t*-test (^&^ indicates a significant difference versus the CH group); SEM, pooled standard error of mean (standard deviation for all rats divided by the square root of rat number, *n* = 100). SOD, superoxide dismutase; CAT, catalase; Cp, ceruroplasmin; TAS, total antioxidant capacity; MDA, malondialdehyde; 3-NT, 3-nitrotyrosine; PC, protein carbonyl derivatives; 8-OHdG, 8-hydroxy-2′-deoxyguanosine; APE-1, apurinic/apyrimidinic endonuclease 1; OGG1, 8-oxoguanine glycosylase; Casp3, caspase 3; Casp8, caspase 8.

## Data Availability

The original contributions presented in this study are included in the article and Supplementary Materials. Further inquiries can be directed to the corresponding authors.

## Supplementary Materials

The following supporting information can be downloaded at: https://www.mdpi.com/article/10.3390/antiox14060695/s1, Table S1. Level of malondialdehyde (MDA; µmol/g) in selected tissues in rats fed experimental diets; Table S2. Activity of superoxide dismutase (SOD; U/g) in selected tissues in rats fed experimental diets; Table S3. Activity of catalase (CAT; U/g) in selected tissues in rats fed experimental diets.

## Abbreviations

3-NT	3-nitrotyrosine
8-OHdG	8-hydroxy-2′-deoxyguanosine
APE-1	Apurinic/apyrimidinic endonuclease 1
Casp3	Caspase 3
Casp8	Caspase 8
CAT	Catalase
Cp	Ceruloplasmin
CuNPs	Copper nanoparticles
EDTA	Ethylenediaminetetraacetic acid
Group C	Rats fed a control diet with standard Cu content in the mineral mixture (6.5 mg/kg from CuCO3), with 8% of cellulose as a dietary fibre source
Group CH	Rats fed a control diet with enhanced Cu content in the mineral mixture (13 mg/kg from CuCO3), with 8% of cellulose as dietary fibre source
Group CN	Rats fed diets with supplementation of CuNPs (6.5 from Cu-nanoparticles), with 8% of cellulose dietary fibre source
Group CNH	Rats fed diets with supplementation of CuNPs (13 mg/kg from Cu-nanoparticles), with 8% of cellulose dietary fibre source
Group JN	Rats fed diets with supplementation of CuNPs (6.5 mg/kg from Cu-nanoparticles), with 2% of cellulose and 6% of inulin dietary fibre source
Group JNH	Rats fed diets with supplementation of CuNPs (13 mg/kg from Cu-nanoparticles), with 2% of cellulose and 6% of inulin dietary fibre source
Group PN	Rats fed diets with supplementation of CuNPs (6.5 mg/kg from Cu-nanoparticles), with 2% of cellulose and 6% of pectin dietary fibre source
Group PNH	Rats fed diets with supplementation of CuNPs (13 mg/kg from Cu-nanoparticles), with 2% of cellulose and 6% of pectin dietary fibre source
Group SN	Rats fed diets with supplementation of CuNPs (6.5 mg/kg from Cu-nanoparticles), with 2% of cellulose and 6% of psyllium dietary fibre source
Group SNH	Rats fed diets with supplementation of CuNPs (13 mg/kg from Cu-nanoparticles), with 2% of cellulose and 6% of psyllium dietary fibre source
MDA	Malondialdehyde
OGG1	8-oxoguanine DNA glycosylase
PBS	Phosphate-buffered saline
PC	Protein carbonyl derivative
SCFAs	Short-chain fatty acids
SOD	Superoxide dismutase
TAS	Total antioxidant capacity
