# Supplementary material for: Inulin Improves the Redox Response in Rats Fed a Diet Containing Recommended Copper Nanoparticle (CuNPs) Levels, While Pectin or Psyllium in Rats Receive Excessive CuNPs Levels in the Diet
Source: Antioxidants (Basel). 2025 Jun 8;14(6):695. doi: 10.3390/antiox14060695 (PMC12189630; doi:10.3390/antiox14060695)
Supplement: Supplementary file 1 [file antioxidants-14-00695-s001.zip › Supplementary Materials Table S1.pdf]

Table S1. Level of malondialdehyde (MDA; μmol/g) in selected tissues in rats fed experimental diets (n=10 per group)*.

	Heart	Lungs	Jejunum	Liver	Pancreas	Kidneys	Spleen	Testes
Control C	8.00	5.29	13.4	8.45	6.17	9.25	10.6	0.820
Control CH	7.48	5.31	13.9	4.00	4.65	10.6	10.8	1.09
2-way ANOVA:								
CN	9.75	5.39^{bcd}	7.73$^{b\,\#}$	6.29$^{d\,\#}$	7.14^{bc}	8.59^{bc}	11.6^{bc}	5.79$^{a\,\#}$
CNH	8.66	7.31$^{a\,\&}$	13.4^{a}	4.62^{e}	11.6$^{a\,\&}$	12.0^{a}	9.07^{cd}	5.57$^{a\,\&}$
PN	11.3$^{\#}$	7.11$^{ab\,\#}$	11.9^{a}	4.80$^{e\,\#}$	7.98^{b}	11.6$^{a\,\#}$	19.6$^{a\,\#}$	5.95$^{a\,\#}$
PNH	7.93	6.25^{abc}	12.3$^{a\,\&}$	15.7$^{a\,\&}$	6.89$^{c\,\&}$	11.7^{a}	13.4^{b}	5.76$^{a\,\&}$
JN	5.35$^{\#}$	2.43$^{e\,\#}$	9.31$^{b\,\#}$	5.77$^{de\,\#}$	3.98$^{e\,\#}$	7.43^{c}	7.44$^{cd\,\#}$	0.664^{c}
JNH	3.90$^{\&}$	5.88^{bc}	9.32$^{b\,\&}$	8.55$^{c\,\&}$	5.02^{d}	10.0^{ab}	11.4^{bc}	2.88$^{b\,\&}$
SN	7.72	6.47^{abc}	8.90$^{b\,\#}$	12.2$^{b\,\#}$	6.99^{c}	10.0^{ab}	17.8$^{a\,\#}$	6.70$^{a\,\#}$
SNH	7.53	4.54^{d}	12.7^{a}	6.70$^{d\,\&}$	5.15^{d}	10.1^{ab}	13.7$^{b\,\&}$	3.13$^{b\,\&}$
SEM	*0.304*	*0.181*	*0.292*	*0.376*	*0.241*	*0.244*	*0.503*	*0.270*
CuNPs dose (D)								
L (6.5 mg/kg)	8.54	5.35	9.45	7.27	6.52	9.42	14.1	4.78
H (13 mg/kg)	7.00	5.99	11.9	8.89	7.17	11.0	11.9	4.34
P value	*0.720*	*0.030*	*<0.001*	*<0.001*	*<0.001*	*<0.001*	*<0.001*	*0.243*
Fibre type (F)								
C (cellulose)	9.20^{ab}	6.35	10.6	5.46	9.39	10.3	10.3	5.68
P (pectin)	9.64^{a}	6.68	12.1	10.2	7.43	11.7	16.5	5.85
J (inulin)	4.62^{c}	4.16	9.32	7.16	4.50	8.72	9.42	1.77
S (psyllium)	7.63^{b}	5.50	10.8	9.47	6.07	10.1	15.8	4.91
P value	*<0.001*	*<0.001*	*<0.001*	*<0.001*	*<0.001*	*<0.001*	*<0.001*	*<0.001*
Interaction D×F								
P value	*0.206*	*<0.001*	*<0.001*	*<0.001*	*<0.001*	*0.020*	*0.001*	*<0.001*

 https://doi.org/10.3390/antiox14060695

*The dietary treatments used in the experimental feeding period: groups C and CH, fed a control diet with standard and enhanced Cu content in the mineral mixture (6.5 and 13 mg/kg from $CuCO_3$, respectively) with 8% of cellulose as dietary fibre source; groups CN and CNH, fed diets with supplementation of CuNPs (6.5 and 13 mg/kg from Cu-nanoparticles, respectively) with 8% of cellulose dietary fibre source; groups PN and PNH, fed diets with supplementation of CuNPs (6.5 and 13 mg/kg from Cu-nanoparticles, respectively) with 2% of cellulose and 6% of pectin dietary fibre source; groups JN and JNH, fed diets with supplementation of CuNPs (6.5 and 13 mg/kg from Cu-nanoparticles, respectively) with 2% of cellulose and 6% of inulin dietary fibre source; groups SN and SNH, fed diets with supplementation of CuNPs (6.5 and 13 mg/kg from Cu-nanoparticles, respectively) with 2% of cellulose and 6% of psyllium dietary fibre source; L, treatment (n=40) with dietary CuNPs 6.5 mg/kg dose; H, treatment (n=40) with dietary CuNPs 13 mg/kg dose; C, treatment (n=20) with cellulose as dietary fibre; P, treatment (n=20) with pectin as dietary fibre; J, treatment (n=20) with inulin as dietary fibre; S, treatment (n=20) with psyllium as dietary fibre; [a-e] Mean values within a column with unlike superscript letters are shown to be significantly different ($P<0.05$); differences among the groups (CN, CNH, PN, PNH, JN, JNH, SN, SNH) are indicated with superscripts only in the case of a statistically significant interaction D×F ($P<0.05$). Additionally, each experimental group fed Cu-NP 6.5 mg/kg (CN, PN, JN, SN) was compared with the control C one with the aid of t-test ([#] indicates a significant difference versus the C group); similarly, each experimental group fed Cu-NP 13 mg/kg (CNH, PNH, JNH, SNH) was compared with the control CH one with the aid of t-test ([&] indicates a significant difference versus the CH group); SEM, pooled standard error of mean (standard deviation for all rats divided by the square root of rat number, n=100).
