# Supplementary material for: Inulin Improves the Redox Response in Rats Fed a Diet Containing Recommended Copper Nanoparticle (CuNPs) Levels, While Pectin or Psyllium in Rats Receive Excessive CuNPs Levels in the Diet
Source: Antioxidants (Basel). 2025 Jun 8;14(6):695. doi: 10.3390/antiox14060695 (PMC12189630; doi:10.3390/antiox14060695)
Supplement: Supplementary file 1 [file antioxidants-14-00695-s001.zip › Supplementary Materials Table S2.pdf]

antioxidants

MDPI

Table S2. Activity of superoxide dismutase (SOD; U/g) in selected tissues in rats fed experimental diets (n=10 per group)*.

	Heart	Lungs	Jejunum	Liver	Pancreas	Kidneys	Spleen	Testes
Control C	2264	536	430	10111	413	1713	1322	1635
Control CH	1802	333	434	9893	555	2496	1345	1554
2-way ANOVA:								
CN	1731$^{b\ \#}$	310$^{de\ \#}$	374$^{d\ \#}$	7963$^{c\ \#}$	431^{cd}	2347$^{b\ \#}$	1264^{a}	1531
CNH	2151$^{a\ \&}$	350^{c}	406^{bc}	10821^{a}	465$^{c\ \&}$	2576^{a}	1350^{a}	1496
PN	2166^{a}	334$^{cd\ \#}$	413^{bc}	10024^{b}	474$^{c\ \#}$	2491$^{a\ \#}$	1425^{a}	1586
PNH	1862^{b}	320^{cde}	396$^{cd\ \&}$	7214$^{d\ \&}$	433$^{cd\ \&}$	2563^{a}	1288^{a}	1513
JN	1757$^{b\ \#}$	753$^{a\ \#}$	456^{a}	9987^{b}	819$^{a\ \#}$	2558$^{a\ \#}$	872$^{b\ \#}$	1470
JNH	1744^{b}	296$^{e\ \&}$	425^{b}	8039$^{c\ \&}$	392$^{d\ \&}$	2522^{a}	1294^{a}	1494
SN	1722$^{b\ \#}$	623^{b}	395^{cd}	7911$^{c\ \#}$	729$^{b\ \#}$	2540$^{a\ \#}$	1345^{a}	1539
SNH	2246$^{a\ \&}$	308$^{de\ \&}$	378$^{d\ \&}$	8129$^{c\ \&}$	402$^{d\ \&}$	2524^{a}	1364^{a}	1547
SEM	*44.308*	*16.336*	*4.255*	*146.008*	*14.952*	*70.017*	*22.540*	*13.737*
CuNPs dose (D)								
L (6.5 mg/kg)	1844	505	410	8971	613	2484	1226	1532
H (13 mg/kg)	2001	319	401	8551	423	2546	1324	1513
P value	*0.011*	*<0.001*	*<0.001*	*0.006*	*0.016*	*<0.001*	*0.019*	*0.426*
Fibre type (F)								
C (cellulose)	1941	330	390	9392	448	2462	1307	1514
P (pectin)	2014	327	404	8619	454	2527	1357	1549
J (inulin)	1751	525	441	9013	606	2540	1083	1482
S (psyllium)	1984	466	387	8020	565	2532	1354	1543
P value	*<0.001*	*<0.001*	*<0.001*	*<0.001*	*<0.001*	*<0.001*	*<0.001*	*0.186*
Interaction D×F								
P value	*<0.001*	*<0.001*	*0.009*	*<0.001*	*<0.001*	*<0.001*	*<0.001*	*0.486*

*The dietary treatments used in the experimental feeding period: groups C and CH, fed a control diet with standard and enhanced Cu content in the mineral mixture (6.5 and 13 mg/kg from $CuCO_3$, respectively) with 8% of cellulose as dietary fibre source; groups CN and CNH, fed diets with supplementation of Cu-NP (6.5 and 13 mg/kg from Cu-nanoparticles, respectively) with 8% of cellulose dietary fibre source; groups PN and PNH, fed diets with supplementation of Cu-NP (6.5 and 13 mg/kg from Cu-nanoparticles, respectively) with 2% of cellulose and 6% of pectin dietary fibre source; groups JN and JNH, fed diets with supplementation of CuNPs (6.5 and 13 mg/kg from Cu-nanoparticles, respectively) with 2% of cellulose and 6% of inulin dietary fibre source; groups SN and SNH, fed diets with supplementation of CuNPs (6.5 and 13 mg/kg from Cu-nanoparticles, respectively) with 2% of cellulose and 6% of psyllium dietary fibre source; L, treatment (n=40) with dietary CuNPs 6.5 mg/kg dose; H, treatment (n=40) with dietary CuNPs 13 mg/kg dose; C, treatment (n=20) with cellulose as dietary fibre; P, treatment (n=20) with pectin as dietary fibre; J, treatment (n=20) with inulin as dietary fibre; S, treatment (n=20) with psyllium as dietary fibre; $^{a\text{-}e}$ Mean values within a column with unlike superscript letters are shown to be significantly different ($P<0.05$); differences among the groups (CN, CNH, PN, PNH, JN, JNH, SN, SNH) are indicated with superscripts only in the case of a statistically significant interaction D×F ($P<0.05$). Additionally, each experimental group fed Cu-NP 6.5 mg/kg (CN, PN, JN, SN) was compared with the control C one with the aid of t-test ($^{\#}$ indicates a significant difference versus the C group); similarly, each experimental group fed Cu-NP 13 mg/kg (CNH, PNH, JNH, SNH) was compared with the control CH one with the aid of t-test ($^{\&}$ indicates a significant difference versus the CH group); SEM, pooled standard error of mean (standard deviation for all rats divided by the square root of rat number, n=100).
