# Supplementary material for: Inulin Improves the Redox Response in Rats Fed a Diet Containing Recommended Copper Nanoparticle (CuNPs) Levels, While Pectin or Psyllium in Rats Receive Excessive CuNPs Levels in the Diet
Source: Antioxidants (Basel). 2025 Jun 8;14(6):695. doi: 10.3390/antiox14060695 (PMC12189630; doi:10.3390/antiox14060695)
Supplement: Supplementary file 1 [file antioxidants-14-00695-s001.zip › Supplementary Materials Table S3.pdf]

Table S3. Activity of catalase (CAT; U/g) in selected tissues in rats fed experimental diets (n=10 per group)*.

	Heart	Lungs	Jejunum	Liver	Pancreas	Kidneys	Spleen	Testes
Control C	73.1	57.8	14.8	686	15.6	406	34.0	11.5
Control CH	63.5	53.3	17.2	705	15.4	179	34.8	8.12
2-way ANOVA:								
CN	89.0$^{a\,\#}$	36.7$^{cd\,\#}$	16.2$^{b\,\#}$	1018$^{bc\,\#}$	23.5$^{a\,\#}$	49.6$^{f\,\#}$	17.4$^{c\,\#}$	11.2^{a}
CNH	41.1$^{d\,\&}$	30.9$^{d\,\&}$	9.71$^{d\,\&}$	441$^{e\,\&}$	9.46$^{d\,\&}$	83.5$^{e\,\&}$	12.9$^{d\,\&}$	4.95$^{d\,\&}$
PN	38.5$^{d\,\#}$	49.1^{b}	4.76$^{e\,\#}$	889$^{c\,\#}$	17.3^{bc}	89.8$^{e\,\#}$	23.2$^{ab\,\#}$	9.55^{ab}
PNH	78.1$^{ab\,\&}$	59.2$^{a\,\&}$	18.3^{a}	553$^{e\,\&}$	15.1^{bc}	126$^{d\,\&}$	26.2^{ab}	9.13$^{b\,\&}$
JN	66.5^{bc}	58.7^{a}	19.8$^{a\,\#}$	940$^{c\,\#}$	13.9^{c}	177$^{b\,\#}$	31.5^{a}	8.11$^{bc\,\#}$
JNH	61.3^{c}	47.1^{b}	20.3$^{a\,\&}$	723^{d}	17.2^{bc}	137$^{cd\,\&}$	26.2^{ab}	8.26^{bc}
SN	64.2^{c}	56.6^{a}	13.4$^{c\,\#}$	1106$^{b\,\#}$	18.1^{b}	148$^{c\,\#}$	34.9^{a}	7.11$^{c\,\#}$
SNH	82.4$^{a\,\&}$	40.6$^{c\,\&}$	18.7^{a}	1622$^{a\,\&}$	14.4^{c}	218$^{a\,\&}$	19.2$^{c\,\&}$	9.70$^{ab\,\&}$
SEM	*2.085*	*1.195*	*0.521*	*35.086*	*0.465*	*9.969*	*4.729*	*0.259*
CuNPs dose (D)								
L (6.5 mg/kg)	64.5	50.3	13.6	988	18.2	116	26.8	8.98
H (13 mg/kg)	65.7	44.4	16.8	835	14.0	141	21.1	8.01
P value	*0.032*	*<0.001*	*0.228*	*0.018*	*<0.001*	*<0.001*	*<0.001*	*0.022*
Fibre type (F)								
C (cellulose)	65.0	33.8	13.0	730	16.5	66.6	15.2	8.06
P (pectin)	58.3	54.1	11.5	721	16.2	108	24.7	9.34
J (inulin)	63.9	52.9	20.1	832	15.5	157	28.8	8.18
S (psyllium)	73.3	48.6	16.1	1364	16.2	183	27.0	8.41
P value	*0.018*	*<0.001*	*<0.001*	*<0.001*	*0.855*	*<0.001*	*<0.001*	*0.131*
Interaction D×F								
P value	*<0.001*	*<0.001*	*<0.001*	*<0.001*	*<0.001*	*<0.001*	*<0.001*	*<0.001*

*The dietary treatments used in the experimental feeding period: groups C and CH, fed a control diet with standard and enhanced Cu content in the mineral mixture (6.5 and 13 mg/kg from $CuCO_3$, respectively) with 8% of cellulose as dietary fibre source; groups CN and CNH, fed diets with supplementation of Cu-NP (6.5 and 13 mg/kg from Cu-nanoparticles, respectively) with 8% of cellulose dietary fibre source; groups PN and PNH, fed diets with supplementation of CuNPs (6.5 and 13 mg/kg from Cu-nanoparticles, respectively) with 2% of cellulose and 6% of pectin dietary fibre source; groups JN and JNH, fed diets with supplementation of CuNPs (6.5 and 13 mg/kg from Cu-nanoparticles, respectively) with 2% of cellulose and 6% of inulin dietary fibre source; groups SN and SNH, fed diets with supplementation of CuNPs (6.5 and 13 mg/kg from Cu-nanoparticles, respectively) with 2% of cellulose and 6% of psyllium dietary fibre source; L, treatment (n=40) with dietary CuNPs 6.5 mg/kg dose; H, treatment (n=40) with dietary CuNPs 13 mg/kg dose; C, treatment (n=20) with cellulose as dietary fibre; P, treatment (n=20) with pectin as dietary fibre; J, treatment (n=20) with inulin as dietary fibre; S, treatment (n=20) with psyllium as dietary fibre; $^{a\text{-}e}$ Mean values within a column with unlike superscript letters are shown to be significantly different ($P<0.05$); differences among the groups (CN, CNH, PN, PNH, JN, JNH, SN, SNH) are indicated with superscripts only in the case of a statistically significant interaction D×F ($P<0.05$). Additionally, each experimental group fed Cu-NP 6.5 mg/kg (CN, PN, JN, SN) was compared with the control C one with the aid of t-test ($^{\#}$ indicates a significant difference versus the C group); similarly, each experimental group fed Cu-NP 13 mg/kg (CNH, PNH, JNH, SNH) was compared with the control CH one with the aid of t-test ($^{\&}$ indicates a significant difference versus the CH group); SEM, pooled standard error of mean (standard deviation for all rats divided by the square root of rat number, n=100).
